# Intensive care scores predict outcomes in patients receiving cytoreductive surgery and hyperthermic intraperitoneal chemotherapy

**DOI:** 10.3389/fsurg.2025.1664710

**Published:** 2025-10-21

**Authors:** Julia Wimmer, Miklos Acs, Gyula Bohus, Patricia Hauer, Veronika Müller, Niklas Bogovic, Paul Kupke, Przemyslaw Slowik, Hans J. Schlitt, Matthias Hornung, Jens M. Werner

**Affiliations:** 1 Department of Anesthesiology, University Medical Center Regensburg, Regensburg, Germany; 2 Department of Surgery, University Medical Center Regensburg, Regensburg, Germany; 3 Faculty of Medicine, Semmelweis University, Budapest, Hungary

**Keywords:** cytoreductive surgery, HIPEC, intensive care unit, long term outcome, SAPS, SOFA

## Abstract

**Introduction:**

Surgical management of patients with peritoneal surface malignancies (PSM) via multivisceral resection is associated with increased morbidity and mortality in the immediate postoperative period, rendering intensive care therapy critically important. We aimed to determine whether intensive care unit (ICU) course and scoring systems predict not only short-term but also long-term outcomes.

**Methods:**

We retrospectively analyzed the medical records of all patients who underwent cytoreductive surgery (CRS) and hyperthermic intraperitoneal chemotherapy (HIPEC) for peritoneal surface malignancies (PSM) between 2008 and 2015 at a university cancer center. Upon postoperative ICU admission, Simplified Acute Physiology Score (SAPS II) and Sequential Organ Failure Assessment (SOFA) scores were recorded. Complications during the ICU stay and overall hospitalization were documented, and patients were followed according to a standardized protocol after discharge.

**Results:**

A total of 251 patients were included. The mean Peritoneal Cancer Index (PCI) was 14 ± 9.1 and correlated significantly with both ICU stay duration (*p* = 0.002) and total hospital stay (*p* = 0.001). In-hospital mortality was 2%, and the reoperation rate was 16.7%. SOFA scores on the day of surgery, postoperative days 1, 2, and 7 demonstrated strong correlations with ICU length of stay (all *p* ≤ 0.001) and with overall hospital stay (*p* = 0.001 for the day of surgery and day 7; *p* ≤ 0.001 for days 1 and 2). In multivariate analysis, SOFA score on postoperative day 7 [hazard ratio (HR) 1.261; 95% confidence interval (CI) 1.120–1.421; *p* ≤ 0.001] and SAPS II on the day of surgery (HR 1.042; 95% CI 1.017–1.068; *p* ≤ 0.001) emerged as independent predictors of overall survival.

**Discussion:**

In conclusion, SAPS II and SOFA scores not only predict ICU and hospital lengths of stay but also independently forecast overall survival in patients undergoing CRS and HIPEC for PSM.

## 1 Introduction

Cytoreductive surgery (CRS), with or without hyperthermic intraperitoneal chemotherapy (HIPEC), has become a curative-intent treatment for selected patients with peritoneal surface malignancies (PSM) of various origins.

Before the introduction of multimodal therapy, the natural course of peritoneal carcinomatosis was reviewed in several studies and consistently showed poor outcomes (1, 2). The foundation of treatment lies in peritonectomy and multivisceral resections, which have been standardized since their original description (3–5). Since then, attention has shifted to the morbidity and mortality associated with this extensive surgery combined with HIPEC, with reported complication rates ranging from 12%–60% and mortality rates between 0.9%–5.8% (6–9).

Due to the complexity and elevated risk of CRS and HIPEC, postoperative admission to the intensive care unit (ICU) has become routine. While substantial data exist on short-term morbidity and mortality, limited information is available regarding the impact of the ICU course on long-term survival. Therefore, the aim of this study was to determine whether intensive care outcome scores—Sequential Organ Failure Assessment (SOFA) and Simplified Acute Physiology Score II (SAPS II)—are predictive not only of in-hospital complications but also of long-term outcomes following CRS and HIPEC.

## 2 Materials and methods

Between September 2008 and March 2015, 251 patients with peritoneal surface malignancies (PSM)—most commonly colorectal, gastric, or ovarian cancer, pseudomyxoma peritonei, or peritoneal mesothelioma—underwent CRS and HIPEC at the Department of Surgery, University Medical Center Regensburg. Data were retrospectively collected from the institutional HIPEC database. Ethical approval was obtained (No. 15-101-038). Inclusion criteria were histologically confirmed peritoneal surface malignancy and treatment with CRS and HIPEC at our institution between 2008 and 2015. Patients were excluded if they had incomplete perioperative data, underwent non-curative/palliative procedures.

### 2.1 Intensive care scores

The SOFA score was first published in 1996 to assess how organ failure contributes to acute morbidity in ICU patients (10). It consists of six different categories—respiratory, nervous, cardiovascular, liver, coagulation and renal—each scored from 0 to 4, with higher values indicating more severe dysfunction (11). The total score is calculated by summing the individual organ scores, resulting in a range from 0 (best) to 24 (worst).

The SAPS II (Simplified Acute Physiology Score) was introduced in 1993 to predict in-hospital mortality in ICU patients (12). It incorporates 17 variables: 12 physiological measures, three related to underlying disease, along with age and reason for ICU admission. Each variable carries a weight from 0 to 26 points, giving a possible total score of up to 163. SAPS II is calculated daily using the most abnormal values recorded within a 24 h period.

Neither score was originally designed to predict long-term survival.

### 2.2 Preoperative evaluation of feasibility for CRS and HIPEC

Eligibility for CRS and HIPEC was assessed using a standardized protocol. Patients underwent medical history, physical examination, ASA and ECOG performance status assessment (13), laboratory testing including tumor markers, and cross-sectional imaging. Contrast-enhanced CT of the abdomen and thorax was the standard modality, occasionally supplemented by MRI for improved preoperative estimation of the Peritoneal Cancer Index (PCI) (14).

However, the intraoperative PCI as determined by the surgeon remained the gold standard. If staging remained uncertain, PET or diagnostic laparoscopy was performed (15).

Indications for CRS and HIPEC included the feasibility of achieving complete or near-complete cytoreduction (CCR0/1) in the absence of extra-abdominal disease. Contraindications were extensive small bowel involvement, unresectable metastases, ECOG ≥ 2, or severe comorbidities.

All cases were reviewed in an interdisciplinary tumor board, followed by anesthesiologic evaluation of the patients ASA-Score (16).

### 2.3 CRS and HIPEC

The primary goal of CRS and HIPEC was complete macroscopic cytoreduction. Tumor burden was documented intraoperatively using the PCI and the Completeness of Cytoreduction (CCR) score (17). All patients received broad-spectrum antibiotics—typically a second-generation cephalosporin plus metronidazole—administered before induction. Re-dosing was performed for prolonged procedures or significant blood loss, and prophylaxis was continued for 24 h postoperatively.

Peritonectomy procedures were carried out according to the technique described by Sugarbaker et al. (3).

HIPEC was performed using the closed-abdomen technique at 42 °C. The following regimens were applied based on tumor histology:

For colorectal and appendiceal carcinoma as well as pseudomyxoma peritonei, 5-fluorouracil (400 mg/m^2^ i.v.), folinic acid (20 mg/m^2^ i.v.), and oxaliplatin (300 mg/m^2^ i.p.) dissolved in 5% dextrose were administered over 30 min. No increase in non-surgical bleeding attributable to the carrier solution was observed. Following the PRODIGE 7 trial (18), mitomycin C replaced the bidirectional FOLFOX regimen and was administered over 60 min.

For gastric and ovarian cancer as well as peritoneal mesothelioma, cisplatin (75 mg/m^2^ i.p.) and doxorubicin (15 mg/m^2^ i.p.) were used for 60 min. To prevent cisplatin-induced nephrotoxicity, all patients received adequate hydration and forced diuresis; sodium thiosulfate was not routinely used.

At the end of the treatment, the abdominal cavity was drained of the chemotherapeutic solution without additional lavage, and the patient was transferred directly to the ICU for further observation.

### 2.4 Postoperative treatment

During admission to the ICU, vitality parameters as well as the SAPS II (Simplified Acute Physiology Score) (12) and the SOFA (Sequential Organ Failure Assessment) (10) scores were determined. These assessments included heart rate, blood pressure, respiratory parameters, and blood tests for kidney and liver function. Neurological bedside tests were performed, and pre-existing conditions were documented. All evaluations were repeated on postoperative days 1, 2, and 7 (Table 1).

**Table 1:** Summary statistics for the simplified acute physiology score II (SAPS II) and the sequential organ failure assessment (SOFA) at predefined perioperative time points (preoperative assessment, ICU admission, postoperative days 1, 2, and 7).

Score Type	SOFA						SAPS					
Measure	Overall	Endpoint = 1	Endpoint = 0	Group comparison p	Survival analysis p	Cox HR [95% CI]	Overall	Endpoint = 1	Endpoint = 0	Group comparison p	Survival analysis p	Cox HR [95% CI]
Preoperative	0.0 [0.0–0.0]	0.0 [0.0–0.0]	0.0 [0.0–0.0]	0.271	0.412	1.07 [0.91–1.26]	N/A	N/A	N/A	N/A	N/A	N/A
ICU Admission	3.0 [0.0–4.0]	2.6 ± 1.9	3.0 [0.0–4.0]	0.282	0.028	1.22 [1.02–1.45]	23.3 ± 7.0	24.4 ± 7.1	21.7 ± 6.5	0.008	<0.001	1.36 [1.15–1.62]
POD 1	1.0 [0.0–3.0]	1.0 [0.0–3.0]	1.0 [0.0–3.0]	0.234	0.172	1.13 [0.95–1.34]	21.0 ± 6.5	21.6 ± 6.7	20.2 ± 6.0	0.097	0.020	1.23 [1.03–1.45]
POD 2	1.0 [0.0–2.0]	1.3 ± 1.7	0.0 [0.0–1.0]	0.008	0.007	1.23 [1.06–1.43]	20.1 ± 5.8	20.9 ± 5.7	16.0 [16.0–21.0]	0.003	0.090	1.14 [0.98–1.34]
POD 7	0.0 [0.0–0.0]	0.0 [0.0–0.2]	0.0 [0.0–0.0]	0.030	<0.001	1.42 [1.21–1.67]	19.0 ± 5.8	19.8 ± 6.2	16.0 [16.0–21.0]	0.045	0.002	1.33 [1.11–1.59]
Preop → ICU Admission	3.0 [0.0–4.0]	3.0 [0.0–4.0]	3.0 [0.0–3.0]	0.362	0.047	1.19 [1.0–1.41]	N/A	N/A	N/A	N/A	N/A	N/A
Preop → POD 7	3.0 ± 1.4	0.0 [0.0–0.0]	0.1 ± 0.8	0.398	<0.001	1.45 [1.21–1.74]	N/A	N/A	N/A	N/A	N/A	N/A
ICU Admission → POD 7	−3.0 [–3.0–0.0]	−3.0 [–3.0–0.0]	−3.0 [–3.0–0.0]	0.733	0.735	0.97 [0.8–1.17]	−4.2 ± 5.5	−4.6 ± 6.1	−3.7 ± 4.4	0.145	0.096	0.85 [0.71–1.03]

For each score (including total and organ-specific components where applicable), the table presents the mean ± SD or median [IQR] for survivors and non-survivors, results of group comparison tests with corresponding *p*-values, and univariable Cox hazard ratios (HR) with 95% confidence intervals (CI) indicating the association between each score and time-to-death. Statistical significance was defined as *p* < 0.05 (two-sided).

If there was clinical suspicion of a pulmonary embolism or an abdominal focus of infection, a CT scan was performed, and the patient was treated accordingly. All patients received prophylactic treatment with unfractionated heparin to prevent thrombosis.

Complications during the ICU stay and the overall hospital stay were documented and later classified according to the Dindo-Clavien classification (19, 20).

After discharge, patients proceeded to rehabilitation and entered a structured follow-up program with visits every three months initially and later every six months. Follow-up included history, physical examination, tumor markers, abdominal ultrasound, and contrast-enhanced CT of the thorax and abdomen.

### 2.5 Data collection and statistical analysis

All data included in this study were collected retrospectively. The following variables were documented for each patient: comorbidities, duration of surgery, extent of resection, transfusion requirements, PCI, CCR status, postoperative morbidity and mortality, and long-term survival. Data sources included surgical and pathology reports, laboratory results, and ICU records. All complications and postoperative survival data were collected up to June 27th, 2017.

#### 2.5.1 Data preparation

All variables were classified as binary, categorical, continuous, or date-time. Normality was tested using the Shapiro–Wilk and D'Agostino–Pearson omnibus tests, supported by a symmetry index (∣mean−median∣/SD < 0.10).

Outliers were identified using a distribution-adaptive approach: for nearly symmetric data, values with an absolute Z-score greater than three were excluded, while for skewed distributions, observations beyond 1.5 × IQR from the first or third quartile were removed. In datasets with more than fifty observations, pruning was limited to the ten most extreme values per variable to preserve statistical power.

#### 2.5.2 Descriptive statistics

Normally distributed variables are presented as mean ± SD; non-normally distributed variables as median [IQR]. Categorical variables are reported as absolute frequencies (%). For clarity, *p*-values below 0.001 are reported as “<0.001”, and statistical significance was defined at a two-sided α-level of 0.05. All descriptive analyses were conducted using the Python packages scipy and lifelines.

#### 2.5.3 Comparative statistics

Associations among categorical variables were assessed using the *χ*^2^ test; Fisher's exact test was used when any expected cell count was below five. Comparisons between continuous variables and binary outcomes were performed using the Mann–Whitney *U* test, as most clinical variables did not follow a Gaussian distribution. Bivariate correlations between continuous variables were analyzed using Pearson's correlation coefficient when both variables were approximately normally distributed; otherwise, Spearman's rank correlation coefficient was used.

Univariate survival analysis was conducted using the Cox proportional hazards model to estimate hazard ratios (HR) with 95% confidence intervals. Proportional hazards assumptions were tested using log-minus-log survival plots and Schoenfeld residuals.

## 3 Results

### 3.1 Baseline characteristics

A total of 251 patients were retrospectively included in the current study. All patients were treated with CRS and HIPEC at the Department of Surgery, University Hospital Regensburg, between September 2008 and March 2015. Of the included patients, 116 (46.2%) were male and 135 (53.8%) female. The mean age was 54 years (±12.7).

The most common origin of peritoneal carcinomatosis was colorectal cancer (*n* = 74), followed by appendiceal origin (*n* = 38), pseudomyxoma peritonei (*n* = 35), gastric cancer (*n* = 25), ovarian cancer (*n* = 36), and mesothelioma (*n* = 23). In 20 cases, the tumor belonged to a rarer subtype or the primary origin could not be identified, consistent with cancer of unknown primary (CUP) syndrome.

Baseline characteristics of the patients are shown in Table 2.

**Table 2:** Baseline characteristics of the study population.

Patient characteristics	Total (*n* = 251)	Percentage (%)
Age (years)	54.0 (±12.7)	–
Sex (m/f)	135/116	53.8/46.2
BMI (kg/m^3^)	25.7 (±4.8)	–
ASA Score		
1	16	6.3
2	165	65.7
±3	70	28
ECOG Score		
0	163	64.9
1	73	29
2	13	5.1
±3	1	0.3
Histology		
Colorectal origin	74	29.5
Appendiceal origin plus high-grade neoplasm	38	15.1
Pseudomyxoma peritonei plus LAMN	35	13.9
Gastric cancer	25	10.0
Ovarian cancer	36	14.3
Mesothelioma	23	9.2
Others	20	8.0
Comorbidities		
Hypertension	73	29.1
Ascites	65	25.9
Diabetes mellitus	18	7.2
Lung disease	18	7.2
Cardiovascular	5	2
Renal	2	0.8

BMI, body mass index; ASA, American Society of Anesthesiologists; ECOG, eastern cooperative oncology group; LAMN, low grade mucinous neoplasm of appendix.

There was no statistically significant difference in performance scores (ASA and ECOG) across the different histological subtypes. The most common classifications were ASA 2 (*n* = 165; 65.7%) and ECOG 0 (*n* = 163; 64.9%).

In 85 patients (33.7%), CRS and HIPEC were performed as a primary treatment; 166 patients (65.9%) had received systemic oncological therapy prior to surgery. In 115 cases (45.6%), the primary tumor had already been resected, and peritoneal carcinomatosis developed metachronously. In 136 patients (54%), the primary tumor and synchronous peritoneal metastases were resected during the CRS and HIPEC procedure.

Twenty patients had “other” tumor types, including: carcinosarcoma (*n* = 1), small bowel carcinoma (*n* = 4), CUP syndrome (*n* = 7), cervical carcinoma (*n* = 1), borderline ovarian tumor (*n* = 1), desmoplastic small round cell tumor (*n* = 1), primary peritoneal carcinoma (*n* = 4), and duodenal carcinoma (*n* = 1).

The most common comorbidity in our patient group was hypertension (*n* = 73; 29.1%), followed by ascites (*n* = 65; 25.9%). Eighteen patients (7.2%) had diabetes mellitus and/or a pulmonary comorbidity. Cardiovascular comorbidities other than hypertension were present in only 5 patients (2%), and renal comorbidities were observed in 2 patients (0.8%).

Tumor staging was determined according to oncological guidelines specific to each tumor histology. Across all histologies, the majority of patients had a T3 tumor stage (62.3%). Approximately 40% had no lymph node metastasis (N0), while 32.7% had N1 disease and 27.7% had N2 or higher. About 20% of patients had no distant metastasis beyond lymph nodes, whereas 79.6% had metastases to other organs.

Only 10% of patients had well-differentiated tumors. Most had either moderately (49.5%) or poorly (40.4%) differentiated tumor grading.

In 184 patients (73.2%), a complete macroscopic tumor resection (CCR0) was achieved. Minimal residual disease (CCR1) was present in 35 patients (14.1%), while complete surgical resection was not possible or not medically indicated in 32 patients (12.7%), resulting in CCR2 classification.

During CRS and HIPEC, the intraoperative peritoneal carcinoma index (PCI) was determined. The mean PCI was 14 ± 9.1. There was a statistically significant difference in PCI between histological subgroups (*p* = 0.017). Higher PCI scores were observed in tumors originating within the peritoneum, such as pseudomyxoma peritonei and peritoneal mesothelioma, while lower scores were found in cases of peritoneal carcinomatosis from colorectal or gastric origin.

Out of the 251 patients, 216 (86.1%) underwent multivisceral resection. The most frequently resected organ was the colon (*n* = 159; 63.3%), followed by the rectum (*n* = 101; 40.2%) and the small bowel (*n* = 83; 31.1%). Liver resections were performed in 44 patients (17.5%), and 89 patients (35.5%) underwent splenectomy.

The mean surgery time was 327 min with a standard deviation of 150 min. The average number of anastomoses was 1 (±1.1). Fifty patients (19.9%) received a blood transfusion during the operation, and 44 patients (17.5%) received ostomy formation.

As perioperative factors potentially influencing the outcome of CRS and HIPEC, the following markers were analyzed: ASA and ECOG scores, the intraoperatively determined PCI, CCR status, type and extent of resection, operation time, intraoperative blood transfusion, development of severe complications according to the Dindo-Clavien-classification (20), as well as the SAPS II and SOFA scores on the day of ICU admission and on postoperative days one, two, and seven. Additionally, the duration of ICU and total hospital stay was recorded.

### 3.2 Perioperative course

On univariable analysis, higher SAPS II values at ICU admission were associated with an increased hazard of death (HR 1.36, 95% CI 1.15–1.62; survival-analysis *p* < 0.001; group-comparison *p* = 0.008). The corresponding SOFA score at ICU admission was also significantly associated with overall survival (HR 1.22, 95% CI 1.02–1.45; *p* = 0.028).

At later postoperative time points, both scores continued to show prognostic value. On postoperative day 2, the SOFA score was associated with a hazard ratio of 1.23 (95% CI 1.06–1.43; *p* = 0.007), and by postoperative day 7, the association strengthened (HR 1.42, 95% CI 1.21–1.67; *p* < 0.001). For SAPS II, significant associations were observed on postoperative day 1 (HR 1.23, 95% CI 1.03–1.45; *p* = 0.020) and again on day 7 (HR 1.33, 95% CI 1.11–1.59; *p* = 0.002).

When examining changes in scores over time, an increase from the preoperative baseline to ICU admission was already predictive of poorer outcomes (HR 1.19, 95% CI 1.00–1.41; *p* = 0.047). A greater rise from baseline to postoperative day 7 was even more strongly associated with decreased survival (HR 1.45, 95% CI 1.21–1.74; *p* < 0.001).

There was no statistically significant correlation between the tumor origin of peritoneal surface malignancy (PSM) and the duration of ICU stay or overall hospital stay (*p* = 0.276 and *p* = 0.722, respectively).

Regardless of tumor origin, the peritoneal carcinoma index (PCI) showed a statistically significant association with both ICU and hospital stay duration (*p* = 0.002 and *p* = 0.001, respectively). Patients with a lower PCI experienced significantly shorter ICU and overall hospital stays.

Among patients in whom a CCR 0/1 status was achieved intraoperatively, there was a statistically significant correlation between PCI and the development of complications (*p* ≤ 0.001). This association was not observed in patients where CCR 0/1 status could not be achieved (*p* = 0.170).

With respect to the duration of ICU stay, a statistically significant correlation was found for patients who underwent multivisceral resection (*p* = 0.008), splenectomy (*p* ≤ 0.001), gastrectomy (*p* ≤ 0.001), and small bowel resection (*p* = 0.011). In contrast, no significant association was observed for colon resection (*p* = 0.187), rectum resection (*p* = 0.924), or liver resection (*p* = 0.102).

A similar pattern was seen for the overall hospital stay. Statistically significant correlations were found with multivisceral resection (*p* = 0.025), splenectomy (*p* = 0.001), small bowel resection (*p* = 0.004), colon resection (*p* = 0.011), and rectum resection (*p* = 0.036). However, there was no significant association between overall hospital stay and gastrectomy (*p* = 0.325) or liver resection (*p* = 0.815).

### 3.3 Complications

Five patients (2%) of the 251 included in the study died during the initial hospital stay. Additionally, 42 patients (16.7%) required at least one reoperation during the same period. The most frequent indication for relaparotomy was anastomotic insufficiency, followed by active bleeding or hematoma formation.

Postoperative complications and their classification are detailed in Tables 3A,B.

**Table 3A:** Postoperative complications.

Complications	Number of patients (%)
Anastomosis insufficiency	11 (4.4%)
Active bleeding or hematoma	7 (2.8%)
Insufficiency of the abdominal wall/fascia	5 (2.0%)
Abscess	4 (1.6%)
Surgery site infection	4 (1.6%)
Bile leakage	3 (1.2%)
Perforation	2 (0.8%)
Compartment syndrome	2 (0.8%)
Exploration without insufficiency	2 (0.8%)
Planned second look	2 (0.8%)

**Table 3B:** Classification of complications according to Clavien-Dindo.

Classification according to Dindo-Clavien	*n* (%)
I	64 (25.7)
II	80 (31.7)
III	89 (35.5)
IV	13 (5.2)
V	5 (2.0)

For revision laparotomy, significant risk estimates were found only for the SOFA score. The preoperative SOFA score was associated with a higher likelihood of reoperation (HR 1.25, 95% CI 1.00–1.56; *p* = 0.047; group-comparison *p* = 0.032). Elevated SOFA scores on postoperative day 1 (HR 1.64, 95% CI 1.24–2.15; *p* < 0.001; group *p* < 0.001), day 2 (HR 1.57, 95% CI 1.22–2.00; *p* < 0.001; group *p* = 0.041), and day 7 (HR 1.32, 95% CI 1.10–1.58; *p* = 0.003; group *p* = 0.043) were each significantly associated with the need for reoperation. A rising SOFA score from baseline to day 7 also showed a significant association (HR 1.26, 95% CI 1.02–1.56; *p* = 0.030). In contrast, none of the SAPS II measurements reached statistical significance for predicting reoperation (all *p* ≥ 0.12).

Furthermore, there was a highly significant correlation between PCI scores and the severity of postoperative complications (*p* ≤ 0.001). Subgroup analysis of patients with a PCI of 20 or higher also revealed a significant association with the development of complications (*p* = 0.001).

The performance of a multivisceral resection was likewise significantly associated with an increased risk of postoperative complications (*p* = 0.002).

Additionally, only splenectomy, gastrectomy, and rectum resection showed statistically significant differences in the distribution of complication severity compared to patients who did not undergo these procedures (*p* ≤ 0.001, *p* = 0.001, and *p* = 0.001, respectively). No significant differences were found for colon resection (*p* = 0.062), small intestine resection (*p* = 0.074), or liver resection (*p* = 0.686).

Of the 246 patients who were discharged from the hospital, 24 (9.8%) were readmitted due to complications during the follow-up period.

### 3.4 Prognostic value of ICU scores for short- and long-term outcome

Both scores reflect the postoperative progression of the patient's condition, as shown in Table 4.

**Table 4:** SOFA and SAPS II-scores.

Day	SOFA	SAPS
0	2.5 ± 2	23.3 ± 7.0
1	1.7 ± 1.6	21.5 ± 6.5
2	1.1 ± 1.5	20.0 ± 5.8
7	0.4 ± 1.3	19.0 ± 5.8

SOFA, sequential organ failure assessment; SAPS, simplified acute physiology score.

A correlation analysis was performed between the two ICU scores and the duration of ICU and overall hospital stay. There was a highly significant correlation between the length of ICU stay and the SOFA scores recorded on the day of surgery (*p* ≤ 0.001), as well as on postoperative days 1, 2, and 7 (all *p* ≤ 0.001). A similar correlation was observed between the SOFA scores and the duration of the overall hospital stay, with significance levels of *p* = 0.001 on the day of surgery, *p* ≤ 0.001 on days 1 and 2, and *p* = 0.001 on day 7.

There was a significant correlation between the repeatedly determined SAPS II scores and both the duration of ICU stay and overall hospital stay. Specifically, the SAPS II score on the day of surgery was significantly associated with ICU stay (*p* ≤ 0.001) and overall hospital stay (*p* ≤ 0.001). A similar correlation was observed for the SAPS II score on postoperative day 7 (*p* = 0.047 and *p* = 0.040, respectively). On postoperative day 2, the SAPS II score showed a statistically significant correlation only with the duration of ICU stay (*p* = 0.020). No statistically significant correlations were found for the other SAPS II measurements.

Three SOFA-derived variables showed significant associations in univariate analyses of both overall survival and the need for reoperation. These were the SOFA score on postoperative day 2 (mortality HR 1.23, 95% CI 1.06–1.43; reoperation HR 1.57, 95% CI 1.22–2.00), the score on postoperative day 7 (mortality HR 1.42, 95% CI 1.21–1.67; reoperation HR 1.32, 95% CI 1.10–1.58), and the change in score from preoperative baseline to postoperative day 7 (mortality HR 1.45, 95% CI 1.21–1.74; reoperation HR 1.26, 95% CI 1.02–1.56).

**Figure 1:**
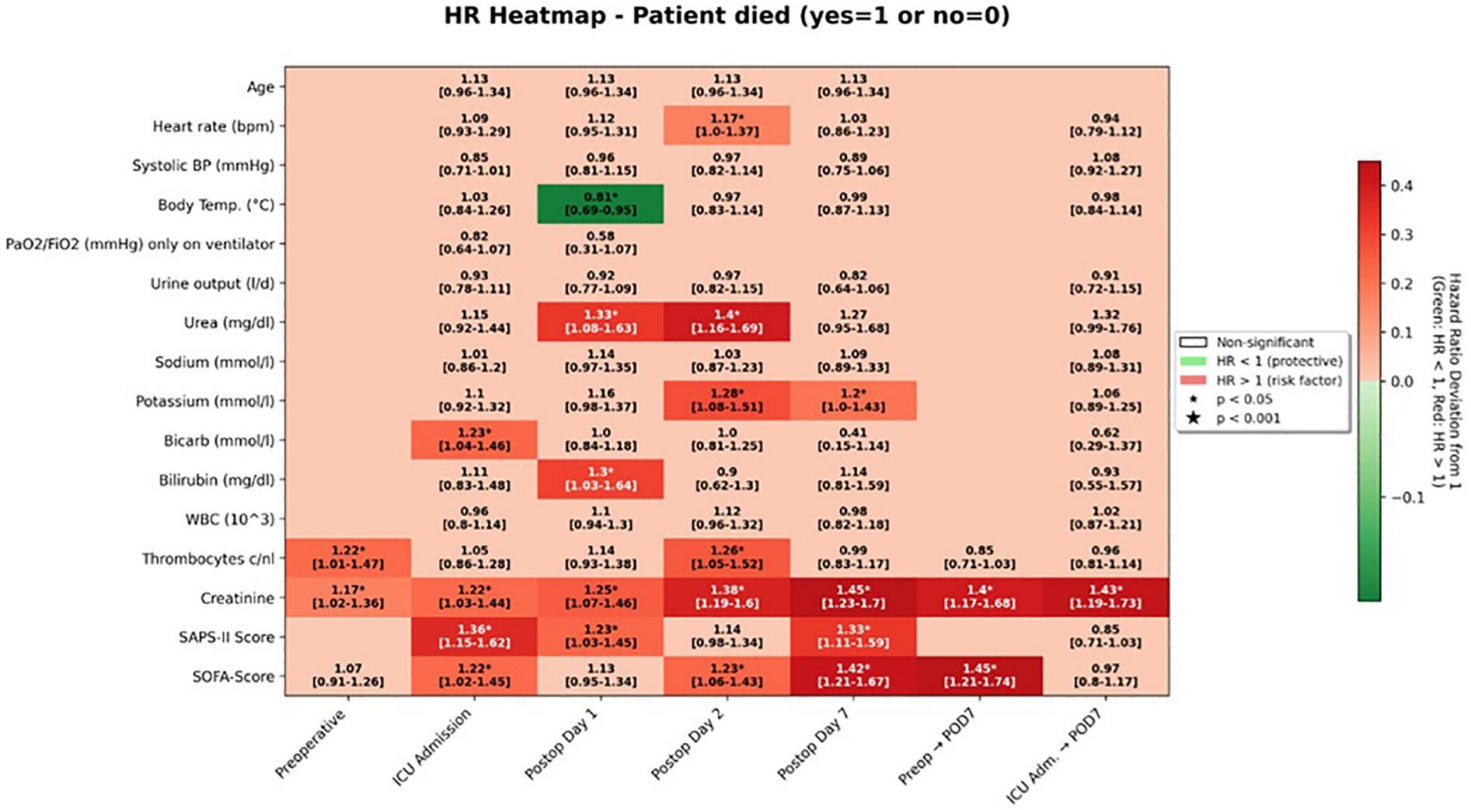
HR heatmap for overall survival.

**Figure 2:**
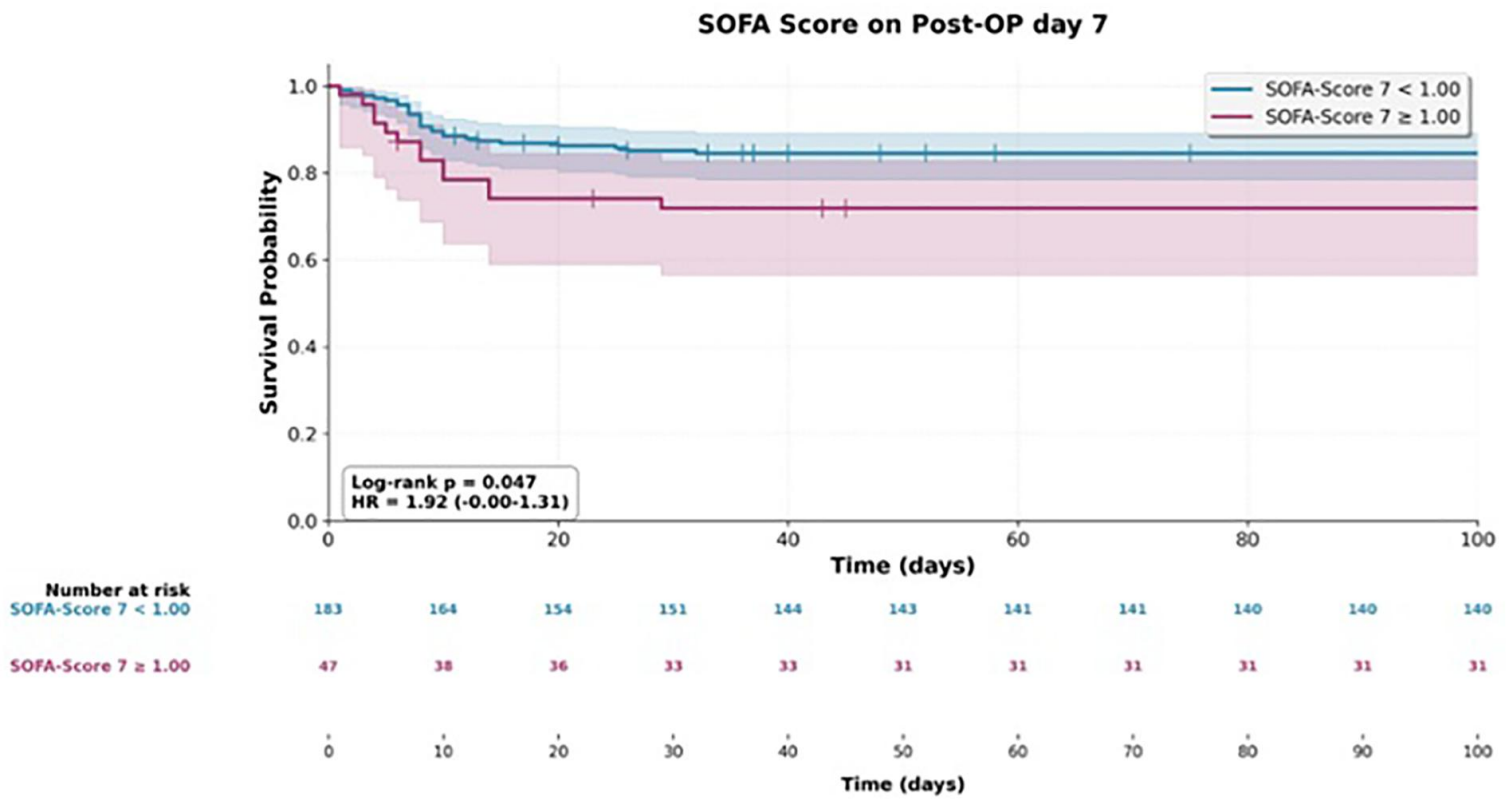
Kaplan–Meier survival distribution stratified by SOFA score on postoperative Day 7.

**Figure 3:**
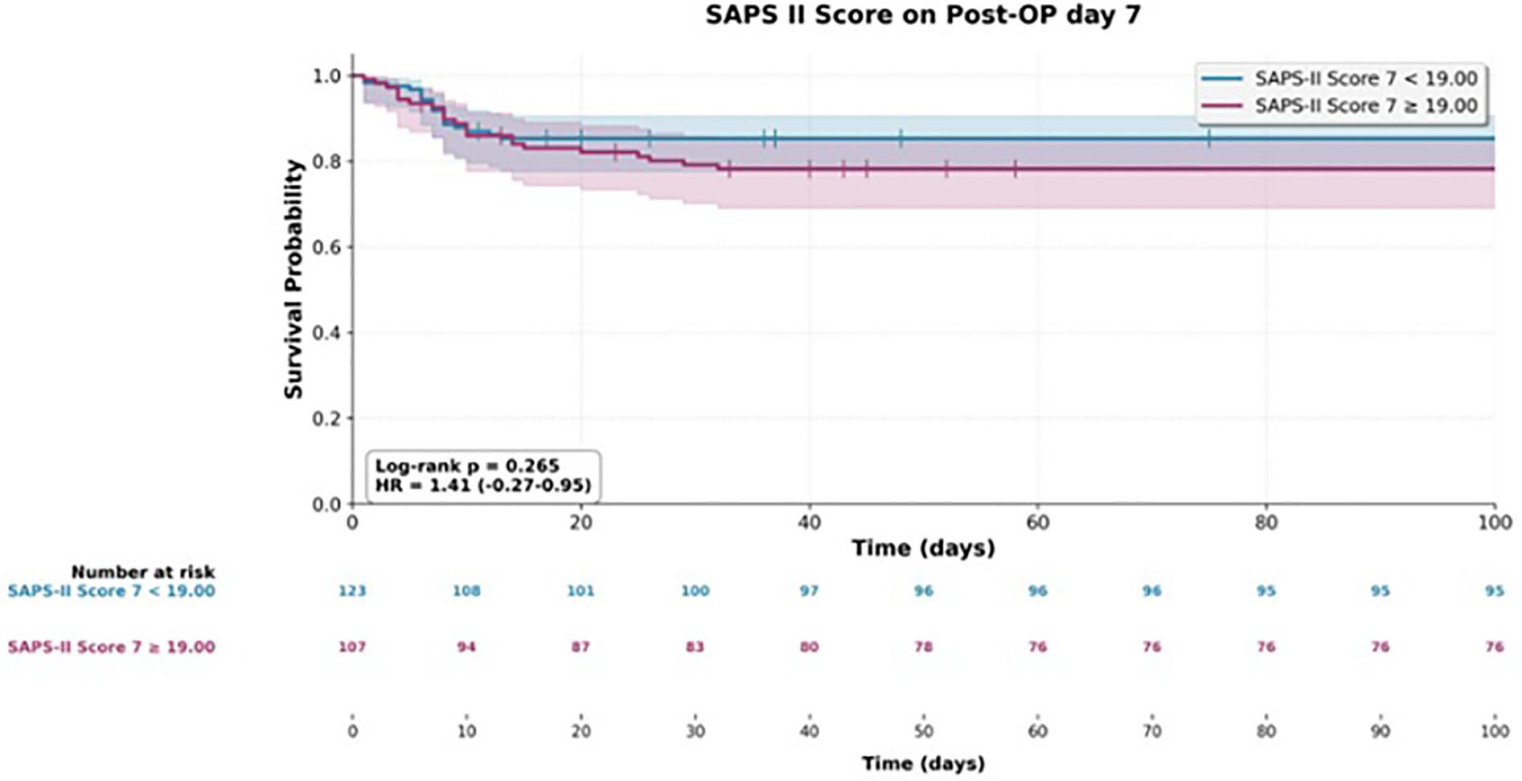
Kaplan–Meier survival distribution stratified by SAPS II score on postoperative Day 7.

In contrast, SAPS II scores yielded significant estimates only in relation to mortality and showed no overlap with findings related to reoperation (Figures 1–3).

**Figure 4:**
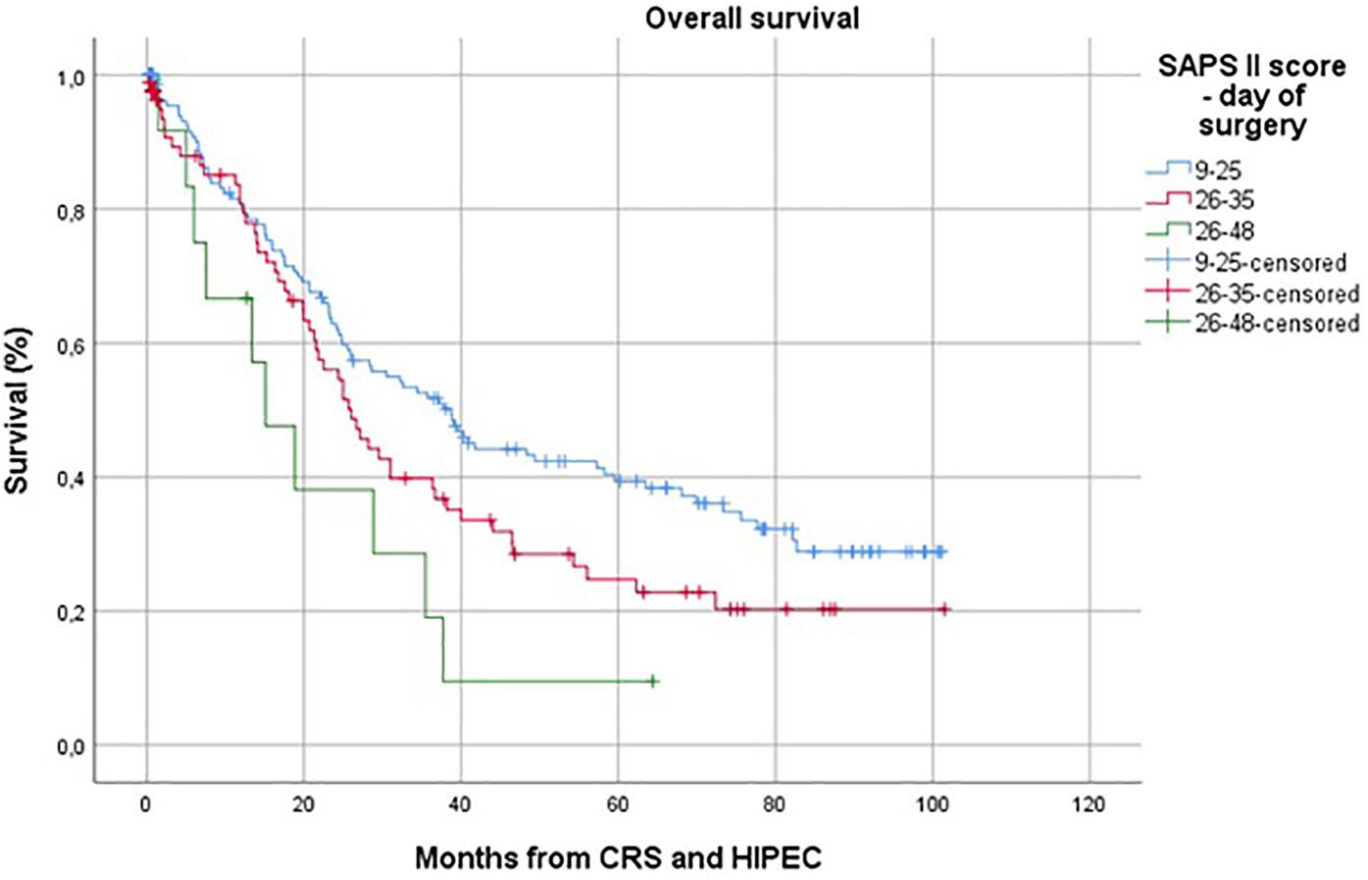
Kaplan–Meier survival distribution stratified by SAPS II score on Day of surgery.

In multivariate analysis, both the SOFA score on postoperative day 7 and the SAPS II score on the day of surgery were identified as independent predictive factors for overall survival. The adjusted hazard ratio for the SOFA score was 1.26 (95% CI 1.12–1.42; *p* ≤ 0.001), and for the SAPS II score, it was 1.04 (95% CI 1.02–1.07; *p* ≤ 0.001) (Figure 4).

The SOFA and SAPS II scores determined on other postoperative days did not serve as predictive factors for overall survival.

In addition to these findings, the preoperatively determined ECOG performance status emerged as a statistically significant prognostic factor (adjusted HR 1.40; 95% CI 1.05–1.86; *p* = 0.022).

The performance of a liver resection during CRS and HIPEC was also associated with a significantly increased hazard of death (adjusted HR 1.93; 95% CI 1.26–2.97; *p* = 0.003).

Finally, achieving complete macroscopic cytoreduction (CCR0) was identified as a favorable prognostic factor for long-term overall survival (adjusted HR 0.66; 95% CI 0.45–0.98; *p* = 0.039).

## 4 Discussion

To our knowledge, this is the first study to evaluate the predictive value of ICU scores for both short- and long-term survival in patients undergoing CRS and HIPEC. We observed that repeated measurements of SAPS II scores and the duration of both ICU stay and overall hospital stay (*p* ≤ 0.001 for both). Furthermore, the SOFA score on postoperative day 7 and the SAPS II score on the day of surgery were identified as independent predictive factors for overall survival. A SAPS II score of ≤25 was associated with improved overall survival (adjusted HR 1.26; 95% CI 1.12–1.42; *p* ≤ 0.001 and adjusted HR 1.04; 95% CI 1.02–1.07; *p* ≤ 0.001, respectively). The cumulative hazard ratios observed for these scores highlight that adverse physiology early and ongoing organ dysfunction both independently and additively compromise survival.

Despite the supporting evidence, the determination of the SOFA score on postoperative day seven proved to be problematic. Patients who recovered well and experienced no major complications were often already discharged from the ICU by that time. As a result, certain components of the SOFA score—such as respiratory parameters—could not be measured accurately. This led to an unintended negative selection bias, as the day-seven SOFA score was only available for a subset of patients (22 of 251) with more complicated postoperative courses. Therefore, we consider this particular result to be unreliable.

The SOFA score was originally developed to assess acute morbidity in ICU patients, rather than to predict the effects of new treatment regimens or overall survival (21). Nates et al. (22) demonstrated the utility of the SOFA score in predicting mortality among both medical and surgical cancer patients, showing that it can be applied effectively in critically ill cancer populations. Additional studies have validated the SOFA score in surgical patients (23), trauma patients (24), and pediatric populations (25).

Among the SOFA score components, the Glasgow Coma Scale (GCS) is considered the most subjective and the least reproducible (26). This limitation was evident in our patient cohort and may have introduced potential bias. The timing of postoperative GCS assessment remains a matter of debate. Ideally, sufficient time should elapse following anesthesia to allow for its effects to wear off, and the influence of analgesics—particularly strong opioids—on consciousness levels must be considered. This is especially relevant in cancer patients, who are frequently treated with high doses of morphine derivatives. Given the prolonged operative duration of CRS and HIPEC procedures, initial postoperative GCS assessments may be significantly compromised.

Recently, Wallet and associates (27) reported no impact of prolonged ICU stay or ICU readmission on long-term survival in patients undergoing CRS and HIPEC. While their study was among the first to address outcomes in this specific patient group following intensive care, it lacked quantitative measures of ICU admission and therapy, resulting in findings that contrast with our own regarding long-term prognosis. A German retrospective study examined outcomes in 852 patients who underwent abdominal, thoracic, or vascular surgery followed by extended ICU treatment, defined as an ICU stay of 20 days or more. Follow-up was available for 502 of these patients (28). The study found that patients with ICU stays of 20 days or longer had significantly lower survival rates compared to those treated for less than 20 days (*p* = 0.003). Furthermore, increased SAPS scores at both ICU admission and discharge (*p* = 0.000 for both) were associated with reduced 12-month survival, underscoring the prognostic value of objective ICU scoring systems. However, no specific SAPS II cutoff was proposed in that study.

It is also important to note that after transfer from the ICU to the general ward, vital parameters were not consistently documented. As a result, both the SOFA and SAPS II scores recorded on postoperative day seven must be interpreted with caution, as they were based on limited clinical information. Nonetheless, the SAPS II score obtained on the day of surgery remained a reliable, objective predictor of overall survival.

Many oncologists still approach the CRS and HIPEC procedure with skepticism due to its perceived high morbidity. In a review Metha et al. (29) summarized the most common complications and divided them into four categories: gastrointestinal, pulmonary, hematological, and others. For gastrointestinal complications, reported rates ranged from 4.5% to 19%, which aligns with our findings, including a reoperation rate of 16.7%. The in-house mortality rate in our study was 2%, consistent with figures reported by other high-volume centers (6, 8).

These outcomes support the view that CRS and HIPEC can be considered safe, with relatively low morbidity and mortality, especially when performed in experienced, high-volume centers. In this context, Desantis et al. (30) analyzed the morbidity, mortality, and the oncological outcome of 401 consecutive CRS and HIPEC procedures performed on 356 patients. They reported a mortality rate of 1%, with 50 patients experiencing a total of 271 complications graded III–IV according to the CTCAE NCI 2006 criteria (12.5%).

These results indicate that one of the most pressing challenges in CRS and HIPEC remains the identification of patients who are most likely to benefit from the procedure. This reinforces the importance of careful patient selection, with the goal of avoiding unnecessary or unsuccessful laparotomies (14).

In a 2012 study, Baratti et al. (31) Analyzing data from 426 patients, they identified, through multivariate analysis, four independent risk factors for morbidity: high PCI, a greater number of visceral resections, poor performance status, and a cisplatin dose exceeding 240 mg.

Our findings align with those of Baratti and colleagues. A high PCI, reflecting extensive tumor burden, often necessitates a more complex surgical approach, which increases the likelihood of complications. Additionally, previous work has shown that the delta temperature—the difference between the lowest and highest intraoperative temperatures—was greatest in patients with high PCI and served as a significant predictor of prolonged ICU stay (9).

While a subset of patients received preoperative chemotherapy, including oxaliplatin-based regimens, no clear association with increased perioperative complications was observed in our cohort. This potential source of bias was considered at the beginning of our analysis and was not evident based on the available data and clinical course of patients. However, as this was not formally analyzed, it warrants further investigation in future prospective studies.

Several studies have suggested a significant learning curve associated with CRS and HIPEC procedures (32–34), which may explain why our data show a trend toward lower mortality rates among patients treated later in the study period.

During the later years of this study, elements of ERAS—including early mobilization, early enteral feeding, and multimodal analgesia—were gradually implemented. These measures have been shown to reduce complications, mortality and length of stay (9, 35, 36) and may have influenced outcomes in our later patient cohort.

Moreover, we and other researchers (37, 38) continue to stress the importance of institutional and surgical experience with this complex treatment. In line with our findings, CRS and HIPEC should be performed in high-frequency centers. This recommendation is also supported by current German therapy guidelines for colorectal and gastric cancer (38, 39).

Perioperative decision-making—such as whether a high tumor burden warrants resection and whether complete cytoreduction is achievable—relies heavily on surgical judgment and experience. As the PRODIGE 7 study demonstrated, increased surgeon experience is associated with improved long-term survival outcomes (40).

In our study, liver resection was associated with a statistically significant reduction in overall survival. However, the survival curves did not show a marked difference immediately after surgery. This suggests that the observed difference in overall survival may be partially attributable to a higher overall tumor stage in these patients, rather than the liver resection itself. Previous studies addressing this topic have demonstrated that liver resection during CRS and HIPEC can be performed safely (18, 41).

The limitations of the present study should be addressed. As a single-center analysis, the results may not be fully generalizable to other institutions. Variability in surgical techniques and HIPEC administration protocols exists across centers, and even within our own institution, these methods and chemotherapy regimens have evolved over time. Additionally, because CRS and HIPEC are applied to a wide range of tumor entities, some degree of heterogeneity in the patient population is unavoidable.

Due to the retrospective design of our study, missing scores could not be retrospectively supplemented. Handling of missing values—assuming absent data as normal—may have introduced systematic bias.

One challenge we encountered was the non-standardized manner in which the Glasgow Coma Scale (GCS) was assessed, introducing potential inter-rater variability. To address missing values, we chose to assume all absent data points as normal.

It is also important to note that several components of the SOFA and SAPS II scores may be influenced by the specific characteristics of the HIPEC procedure itself. For instance, the administered chemotherapy may affect laboratory parameters as a side effect. Moreover, the use of hyperthermia means that a body temperature exceeding 40°C upon ICU admission might be appropriate and expected in this context, whereas such temperatures would typically be considered pathological in patients undergoing other major abdominal surgeries.

Despite their limitations, SAPS II and SOFA are widely available, inexpensive, and objective tools. Incorporating these scores into perioperative decision-making could improve risk stratification in CRS and HIPEC. Combining tumor-related indices (PCI, CCR) with ICU-derived physiological scores (SAPS II, SOFA) and baseline performance metrics (ECOG, ASA) may yield a more comprehensive prognostic framework. This integrative approach could assist in patient counseling, perioperative planning, and identification of high-risk individuals who may benefit from intensified monitoring or ERAS-based supportive measures. Importantly, morbidity and mortality after CRS and HIPEC remain substantial, especially in subgroups such as ovarian cancer, as shown by Polom et al., underscoring the need for careful patient selection and balanced risk assessment (42).

## 5 Conclusion

In conclusion, SAPS II and SOFA scores provide prognostic information that complements established surgical and oncological factors in CRS and HIPEC. While SAPS II on the day of surgery appears robust, SOFA on POD 7 is subject to bias and must be interpreted with caution. Future prospective studies are warranted to validate the role of ICU scores within integrated prognostic frameworks and to explore their potential in guiding perioperative management strategies.

Furthermore, our findings demonstrate that CRS and HIPEC is a safe procedure, with morbidity and mortality rates comparable to those of other major abdominal surgeries. As such, this multimodal treatment should be considered for patients with a low predicted mortality risk, as it remains the most promising curative option for individuals with peritoneal metastases.

## Data Availability

The raw data supporting the conclusions of this article will be made available by the authors, without undue reservation.
